# Multiscale simulation and experimental measurements of the elastic response for constructional steel

**DOI:** 10.1038/s41598-022-26594-0

**Published:** 2022-12-24

**Authors:** Yi-Cong Ye, Feng-Yuan Zhao, Cai-Min Huang, Shu-Xin Bai, Qiang Chen

**Affiliations:** 1 Department of Materials Science and Engineering, College of Aerospace Science and Engineering, National University of Defense Technology, Changsha, 410073 China; 2 School of Mechanical and Electrical Engineering, Guilin University of Electronic Technology, Guilin, 541004 China

**Keywords:** Engineering, Physics, Applied physics, Materials science, Structural materials, Theory and computation

## Abstract

The multiscale elastic response to the macroscopic stress was simulated to reveal the multi-scale correlation of elastic properties of the medium carbon steel. Based on the multiscale correlation constitutive equations derived from this constitutive model, the effective elastic constants (EECs) of medium carbon steel are predicted. In addition, the diffraction elastic constants (DECs) of the constituents of the medium carbon steel are also evaluated. And then, the simple in-situ X-ray diffraction experiments were performed for the measurements of DECs and EECs of treated 35CrMo steel during the four-point bending. Compared with the experimental measurements and different existing models, the results demonstrated that the developed constitutive model was in good agreement with the measured values of the EECs and DECs, and that the feasibility and reliability of the constitutive model used to simulate multiscale elastic response could reveal the correlation between the material and its constitutes.

## Introduction

Engineering materials suffered from manufacturing stages before practical application, such as heat treatments^1^, machining operation^2,3^, and so on^4^, which resulted in residual stress because of external mechanical or thermal loads in materials^5^. On the one hand, purposeful introduction of residual stress could improve the mechanical properties of engineering materials^6,7^. On the other hand, unanticipated failure occurred in materials because of stress concentration^8,9^. Accordingly, residual stress could produce either detrimental or beneficial results, and it is of great significance of their characterization and quantification in deep sight of the material research.

Indeed, the assessment of the residual stress and the effects is limited by the lack of comprehensive information revealing the condition in which the residual stress arose^10^. There are two main experimental techniques of residual stress, including mechanical methods that need to destroy the original state of materials^11,12^, , non-destructive testing (NDT) that convert mechanical response into the measurable output^3,13–16^. However, it is necessary of these methods to use the related equations that transformed the measured strain to corresponding residual stress^17,18^ . Because of time-consuming and cost-expensive experimental determination, multitudinous methods for prediction the residual stress in materials have been developed^19^. These numerical methods that aimed to efficient achievement of modern compute essentially required finite element discretization of the inhomogeneous region, such as finite element modeling (FEM)^20^, Mechanics of structure genome^21,22^, Fast Fourier transforms^23^ and so on. Different from the limited description of physical events of the materials by phenomenological model, the analytical approaches that provide a good reference for revealing the intrinsic properties of materials allow to correlate between the overall behavior of composites and the individual constituents, for instance, elasticity solution^24–27^, Eshelby theory^28^ and energy method^29,30^, which is limited by the complex situation and boundary conditions. Morever, traditional micromechanics is designed to obtain the homogenized characterization at the smallest length scale of the materials^31^, unconsciously ignoring the scale correlation of material deformation mechanism^32,33^.

Consequently, a novel micromechanical model was established to predict the elastic response of the treated 35CrMo steel at multiscale, and the correlative constitutive equations of the elastic response was derived to simulate the residual stress of the treated 35CrMo steel and its constituents. The reliable effective elastic constants (EECs) of the treated 35CrMo steel were predicted by this model, including effective elastic modulus $\overline{E }$, effective bulk modulus $\overline{K }$,effective shear modulus $\overline{G }$, effective Poisson’s ratio $\overline{\nu }$, and diffraction elastic constants (DECs) of constituents, *E*_*hkl*_ and $\nu$_*hkl*_, were also obtained. As a contrast, a simple non-destructive method consisting of the X-ray strain measurements and four-point bending was carried out. By comparing the nondestructive results and different micromechanical models, it is applicable of the developed model to predict the elastic properties of the treated 35CrMo steel and is credible to study the association mechanism of the residual stress.

### Simulation of elastic response

In this model, it is assumed that the interface between the constituents of the material is ideal so that the load can be transmitted uniformly, which is necessary for a comprehensive understanding of multiscale elastic response of whole material and its constituents.

The micromechanical relation between the strain and stress can be defined as^34^

1 $${{\varvec{\sigma}}}_{ij}={{\varvec{C}}}_{ijkl}:{{\varvec{\varepsilon}}}_{kl}$$

where ${{\varvec{C}}}_{ij}$ is elastic stiffness of materials and related to elastic compliance ${{\varvec{S}}}_{ij}$, ${{\varvec{C}}}_{ij}:{{\varvec{S}}}_{ij}={{\varvec{I}}}_{ij}$ $\left(i,j,k,l=\mathrm{1,2},\mathrm{3,4}\right)$. Actually, the conversion from the strain to stress in the materials is inherently complicated because of the unknow strain.

Based on the isotropic continuum mechanics of engineering material under the uniaxial stress, **Eq. (****1****)** can be rewritten by the generalized Hooke’s law^10^.

2 $$\frac{{\varepsilon }_{\phi ,\psi }}{{\sigma }_{11}}=\left(\frac{1+\overline{\nu }}{\overline{E} }\right){\mathrm{sin}}^{2}\psi -\left(\frac{\overline{\nu }}{\overline{E} }\right)$$

where the diffraction angles, $\phi$ and $\psi$, are related to the crystal planes of the constituents (*hkl*); The effective elastic constants (EECs) of the material, including $\overline{E }$, $\overline{\nu }$, $\overline{K }$ and $\overline{G }$, are related by the following

3 $$\overline{E }=\frac{9\overline{K }\overline{G} }{3\overline{K }+\overline{G} }, \overline{\nu }=\frac{3\overline{K }-2\overline{G} }{6\overline{K }+2\overline{G} }$$

where $\overline{K }$ and $\overline{G }$ of the composite were predicted by the Mori-Tanaka^35^, based on the Eshelby effective inclusion theory^36^. However, the interaction between the constituents is not reflected in the effective elastic properties of the whole composite, and the multiscale correlation of elastic properties is ignored^32,33^.

Comparing the Eq. (1) and (2), the effective elastic constants (EECs) of the material reveal the deformation mechanism under macroscopic stress, which is of great significance for predicting the deformation behavior at macroscopic scale. However, in practical applications, residual stress in material is usually estimated directly from diffraction strains^37,38^.

The presence of the intergranular stress in the constituents of material made the deduction of residual stress implausible because of scale effect in the diffraction strain measurements^10^. In other words, the elastic response to the macroscopic stress of the material is intrinsically different because of the elastic anisotropy of the constituents.

On the one hand, there is the uniform strain ${\overline{{\varvec{\varepsilon}}} }_{kl}$ in material. The strain of the constituents is related by

4 $$\left(1-f\right){{\varvec{\varepsilon}}}_{kl}^{0}+f{{\varvec{\varepsilon}}}_{kl}^{1}=0$$

*f* is the volume fractions of the reinforcement; ${{\varvec{\varepsilon}}}_{kl}^{0}$ and ${{\varvec{\varepsilon}}}_{kl}^{1}$ are the strain of the matrix and the reinforcement, respectively. And the solid constitutive relations of the constituents are

5a $${{\varvec{\sigma}}}_{ij}^{0}={{\varvec{C}}}_{ijkl}^{0}:\left({{\varvec{\varepsilon}}}_{kl}^{0}+{\overline{{\varvec{\varepsilon}}} }_{kl}\right)$$

5b $${{\varvec{\sigma}}}_{ij}^{1}={{\varvec{C}}}_{ijkl}^{1}:\left({{\varvec{\varepsilon}}}_{kl}^{1}+{\overline{{\varvec{\varepsilon}}} }_{kl}\right)$$

In this equation, ${{\varvec{\sigma}}}_{ij}^{0}$ and ${{\varvec{\sigma}}}_{ij}^{1}$ are the stress of the matrix and the reinforcement. ${{\varvec{C}}}_{ijkl}^{0}$ and ${{\varvec{C}}}_{ijkl}^{1}$ are the elastic stiffness of the matrix and the reinforcement, respectively.

Because of different elastic properties of the matrix and reinforcement, the above equations should be rewritten as the following, according to the Eshelby effective inclusion theory^36^.

6 $${{\varvec{C}}}_{ijkl}^{1}:\left[\left({{\varvec{\varepsilon}}}_{kl}^{0}+{\overline{{\varvec{\varepsilon}}} }_{kl}\right)+\left({{\varvec{\varepsilon}}}_{kl}^{1}-{{\varvec{\varepsilon}}}_{kl}^{0}\right)\right]={{\varvec{C}}}_{ijkl}^{0}:\left[\left({{\varvec{\varepsilon}}}_{kl}^{0}+{\overline{{\varvec{\varepsilon}}} }_{kl}\right)+\left({{\varvec{\varepsilon}}}_{kl}^{1}-{{\varvec{\varepsilon}}}_{kl}^{0}\right)-{{\varvec{\varepsilon}}}_{kl}^{*}\right]$$

where $\left({{\varvec{\varepsilon}}}_{kl}^{1}-{{\varvec{\varepsilon}}}_{kl}^{0}\right)={{\varvec{\varepsilon}}}_{ij}^{^{\prime}}$ is disturbance strain resulted by elastic inhomogeneity of the constituents; ${{\varvec{\varepsilon}}}_{kl}^{*}$ is the inherent strain and related to the ${{\varvec{\varepsilon}}}_{ij}^{^{\prime}}$, i.e. ${{\varvec{\varepsilon}}}_{ij}^{^{\prime}}={{{\varvec{S}}}_{ijkl}^{\mathrm{E}}:{\varvec{\varepsilon}}}_{kl}^{*}$ with Eshelby inclusion tensor ${{\varvec{S}}}_{ijkl}^{\mathrm{E}}$.

Considering the elastic inhomogeneity, the stress of the reinforcement is defined as

7 $${\overline{{\varvec{\sigma}}} }_{ij}^{1}={{\varvec{C}}}_{ijkl}^{0}:\left[\left({{\varvec{\varepsilon}}}_{kl}^{0}+{\overline{{\varvec{\varepsilon}}} }_{kl}\right)-\left({{\varvec{S}}}_{ijkl}^{\mathrm{E}}-{{\varvec{I}}}_{ijkl}\right):{{\varvec{\varepsilon}}}_{kl}^{*}\right]$$

And then, the average stress of the material is expressed as

8 $${\overline{{\varvec{\sigma}}} }_{ij}=\left(1-f\right){{\varvec{\sigma}}}_{ij}^{0}+f{{\varvec{\sigma}}}_{ij}^{1}={{\varvec{C}}}_{ijkl}^{0}:\left({{\varvec{\varepsilon}}}_{kl}^{0}+{\overline{{\varvec{\varepsilon}}} }_{kl}\right)+f{{\varvec{C}}}_{ijkl}^{0}:\left({{\varvec{S}}}_{ijkl}^{\mathrm{E}}-{{\varvec{I}}}_{ijkl}\right):{{\varvec{\varepsilon}}}_{kl}^{*}$$

And then, the effective elastic stiffness of the whole material can be obtained after complex mathematical deduction.

9 $${\overline{{\varvec{C}}} }_{ijkl}={\left\{{{\varvec{C}}}_{ijkl}^{0}-\left({{\varvec{C}}}_{ijkl}^{0}-{{\varvec{C}}}_{ijkl}^{1}\right):\left[{{\varvec{S}}}_{ijkl}^{\mathrm{E}}-f\left({{\varvec{S}}}_{ijkl}^{\mathrm{E}}-{{\varvec{I}}}_{ijkl}\right)\right]\right\}}^{-1}\left[{{\varvec{C}}}_{ijkl}^{0}-\left(1-f\right)\left({{\varvec{C}}}_{ijkl}^{0}-{{\varvec{C}}}_{ijkl}^{1}\right):{{\varvec{S}}}_{ijkl}^{\mathrm{E}}\right]:{{\varvec{S}}}_{ijkl}^{0}$$

where ${{\varvec{S}}}_{ijkl}^{0}$ is the elastic compliance of the matrix and related to the elastic stiffness ${{\varvec{C}}}_{ijkl}^{0}$, ${{\varvec{C}}}_{ijkl}^{0}:{{\varvec{S}}}_{ijkl}^{0}={{\varvec{I}}}_{ijkl}$ $\left(i,j,k,l=\mathrm{1,2},\mathrm{3,4}\right)$.

On the other hand, it is not rational to describe the micromechanical stress response of the constituents by Eq. (2), because of the inherent difference in diffraction strain measurement ${\overline{{\varvec{\varepsilon}}} }^{m}$^32,36^. The elastic response of the constituents is defined as follows

10 $$\frac{{\overline{{\varvec{\varepsilon}}} }^{m}}{{\tilde{\sigma }}_{11}}=\left(\frac{1+{\nu }_{hkl}}{{E}_{hkl}}\right){\mathrm{sin}}^{2}\psi -\left(\frac{{\nu }_{hkl}}{{E}_{hkl}}\right)$$

where ${\overline{{\varvec{\varepsilon}}} }^{m}$ is diffraction strain of the constituents ($m=0$ for the matrix, $m=1$ for the reinforcement); ${\nu }_{hkl}$ and ${E}_{hkl}$ are diffraction elastic constants (DECs) related to the crystal planes (*hkl*) of the constituents.

Based on the uniform elastic behavior of all grains in materials, the stress and strain response were predicted by Reuss^39^ and Voight^40^ in earlier studies, which is not suitable for elastic anisotropic material. Considering the interaction between grains, the diffraction elastic constants (DECs) of the material were evaluated by Kröner^41^ with the self-consistent method^42^. As mention above, the EECs and DECs of the material are different in physical meaning. The micromechanical elastic response of the constituents to the macroscopic stress is the concentrated embodiment of elastic anisotropy and the elastic deformation of the whole material is described by the EECs at macroscopic scale, which depends on the multiscale correlation of the elastic properties at different scales. The purpose of this established multiscale constitutive model is to explore different elastic deformation mechanisms by revealing the correlation between the material and its constituents.

According to Hill’s deduction^43^, the residual stress of the constituents is related to the macroscopic stress

11 $${\overline{{\varvec{\sigma}}} }_{kl}^{m}={{\varvec{B}}}_{ijkl}^{m}:{\overline{{\varvec{\sigma}}} }_{ij}$$

In above equation, ${\overline{{\varvec{\sigma}}} }_{kl}^{m}$ is the residual stress of constituents; ${{\varvec{B}}}_{ijkl}^{m}=\left(\frac{{\overline{K} }_{m}}{\left({\overline{K} }_{m}-\overline{K }\right)\alpha +\overline{K} },\frac{{\overline{G} }_{m}}{\left({\overline{G} }_{m}-\overline{G }\right)\beta +\overline{G} }\right)$ is the concentration tensor of the constituents, in which ${\overline{K} }_{m}$ and ${\overline{G} }_{m}$ are the effective elastic properties of the constituents, and the parameters $\alpha =\frac{1}{3}\frac{1+\overline{\upnu } }{1-\overline{\upnu } }$ and $\beta =\frac{2}{15}\frac{4-5\overline{\upnu } }{1-\overline{\upnu } }$ are related to the effective Poisson's ratio $\overline{\nu }$ of the material in Eq. (3).

Considering the elastic inhomogeneity, the solid constitutive relation of constituents is

12 $${{\varvec{C}}}_{ijkl}^{1}:\left[{\overline{{\varvec{\varepsilon}}} }_{kl}+{{\varvec{\varepsilon}}}_{ij}^{^{\prime}}\right]={{\varvec{C}}}_{ijkl}^{0}:\left[{\overline{{\varvec{\varepsilon}}} }_{kl}+{{\varvec{\varepsilon}}}_{ij}^{^{\prime}}-{{\varvec{\varepsilon}}}_{kl}^{*}\right]$$

Simultaneous Eq. (6), the strain disturbance can be obtained from

13 $${{\varvec{\varepsilon}}}_{ij}^{^{\prime}}={{\varvec{T}}}_{ijkl}:{\overline{{\varvec{\sigma}}} }_{kl}$$

where ${{\varvec{T}}}_{ijkl}={\left({{\varvec{C}}}_{ijkl}^{1}-{{\varvec{C}}}_{ijkl}^{0}+{{\varvec{C}}}_{ijkl}^{0}:{{{\varvec{S}}}_{ijkl}^{E}}^{-1}\right)}^{-1}:\left({{\varvec{I}}}_{ijkl}-{{\varvec{C}}}_{ijkl}^{1}:{{\varvec{S}}}_{ijkl}^{0}\right):{{{\varvec{B}}}_{ijkl}^{1}}^{-1}$ is defined as the crystal interaction tensor. As a result, the strain of the reinforcement ${{\varvec{\varepsilon}}}_{kl}^{1}$ is

14 $${{\varvec{\varepsilon}}}_{kl}^{1}={\overline{{\varvec{\varepsilon}}} }_{kl}+{{\varvec{\varepsilon}}}_{ij}^{^{\prime}}=\left({{\varvec{T}}}_{ijkl}+{\overline{{\varvec{S}}} }_{ijkl}\right):{\overline{{\varvec{\sigma}}} }_{ij}$$

where ${\overline{{\varvec{S}}} }_{ijkl}$ is the effective elastic compliance of the whole material, ${\overline{{\varvec{S}}} }_{ijkl}:{\overline{{\varvec{C}}} }_{ijkl}={{\varvec{I}}}_{ijkl}$.

Then, the diffraction strains of the reinforcement are obtained by the average of the diffraction strain in the three-dimensional direction

15 $${\overline{\varepsilon }}_{kl}^{1}=\frac{{\int }_{0}^{2\pi }{{\varvec{\varepsilon}}}_{kl}^{1}d\theta }{{\int }_{0}^{2\pi }d\theta }$$

Furthermore, diffraction elastic constants (DECs) of the constituents are deduced from the numerical solution of above equation

16 $$\frac{1+{\nu }_{hkl}}{{E}_{hkl}}=\left(\frac{1+\overline{\nu }}{\overline{E} }\right)+\frac{{u}^{2}}{2}\left[{t}_{11}\left(3{u}^{2}-1\right)+{t}_{12}\left(3{v}^{2}-1\right)+{t}_{13}\left(3{w}^{2}-1\right)+3\left({t}_{14}vw+{t}_{15}uw+{t}_{16}uv\right)\right]+\frac{{v}^{2}}{2}\left[{t}_{21}\left(3{u}^{2}-1\right)+{t}_{22}\left(3{v}^{2}-1\right)+{t}_{23}\left(3{w}^{2}-1\right)+3\left({t}_{24}vw+{t}_{25}uw+{t}_{26}uv\right)\right]+\frac{{w}^{2}}{2}\left[{t}_{31}\left(3{u}^{2}-1\right)+{t}_{32}\left(3{v}^{2}-1\right)+{t}_{33}\left(3{w}^{2}-1\right)+3\left({t}_{34}vw+{t}_{35}uw+{t}_{36}uv\right)\right]+\frac{vw}{2}\left[{t}_{41}\left(3{u}^{2}-1\right)+{t}_{42}\left(3{v}^{2}-1\right)+{t}_{43}\left(3{w}^{2}-1\right)+3\left({t}_{44}vw+{t}_{45}uw+{t}_{46}uv\right)\right]+\frac{uw}{2}\left[{t}_{51}\left(3{u}^{2}-1\right)+{t}_{52}\left(3{v}^{2}-1\right)+{t}_{53}\left(3{w}^{2}-1\right)+3\left({t}_{54}vw+{t}_{55}u\mathrm{w}+{t}_{56}uv\right)\right]+\frac{vu}{2}\left[{t}_{61}\left(3{u}^{2}-1\right)+{t}_{62}\left(3{v}^{2}-1\right)+{t}_{63}\left(3{w}^{2}-1\right)+3\left({t}_{64}vw+{t}_{65}uw+{t}_{66}uv\right)\right]$$

17 $$-\frac{{\nu }_{hkl}}{{E}_{hkl}}=-\left(\frac{\overline{\nu }}{\overline{E} }\right) +\frac{{u}^{2}}{2}\left[{t}_{11}\left(1-{u}^{2}\right)+{t}_{12}\left(1-{v}^{2}\right)+{t}_{13}\left(1-{w}^{2}\right)-\left({t}_{14}vw+{t}_{15}uw+{t}_{16}uv\right)\right]+\frac{{v}^{2}}{2}\left[{t}_{21}\left(1-{u}^{2}\right)+{t}_{22}\left(1-{v}^{2}\right)+{t}_{23}\left(1-{w}^{2}\right)-\left({t}_{24}vw+{t}_{25}uw+{t}_{26}uv\right)\right]+\frac{{w}^{2}}{2}\left[{t}_{31}\left(1-{u}^{2}\right)+{t}_{32}\left(1-{v}^{2}\right)+{t}_{33}\left(1-{w}^{2}\right)-\left({t}_{34}vw+{t}_{35}uw+{t}_{36}uv\right)\right]+\frac{vw}{2}\left[{t}_{41}\left(1-{u}^{2}\right)+{t}_{42}\left(1-{v}^{2}\right)+{t}_{43}\left(1-{w}^{2}\right)-\left({t}_{44}vw+{t}_{45}uw+{t}_{46}uv\right)\right]+\frac{uw}{2}\left[{t}_{51}\left(1-{u}^{2}\right)+{t}_{52}\left(1-{v}^{2}\right)+{t}_{53}\left(1-{w}^{2}\right)-\left({t}_{54}vw+{t}_{55}uw+{t}_{56}uv\right)\right]+\frac{vu}{2}\left[{t}_{61}\left(1-{u}^{2}\right)+{t}_{62}\left(1-{v}^{2}\right)+{t}_{63}\left(1-{w}^{2}\right)-\left({t}_{64}vw+{t}_{65}uw+{t}_{66}uv\right)\right]$$

As shown in Eq. (13), ${t}_{ab}$ (*a*, *b* = 1,2,3……6) are the components of ${{\varvec{T}}}_{ijkl}$; (*u,v,w*) is the directional cosine of crystal planes (*hkl*).

All in all, the effective elastic constants (EECs) that are defined by the Eq. (2), are average properties of the diffraction elastic constants (DECs) that describe the Eq. (10) for the micromechanical elastic response of constituents to macroscopic stress, which is shown by the Eq. (16) and (17). Morever, the multiscale elastic response actually demonstrates the correlation mechanism between the macroscopical elastic deformation of the whole material and the internal mechanic of the constituents.

### Comparison with experimental measurements

**Figure 1:**
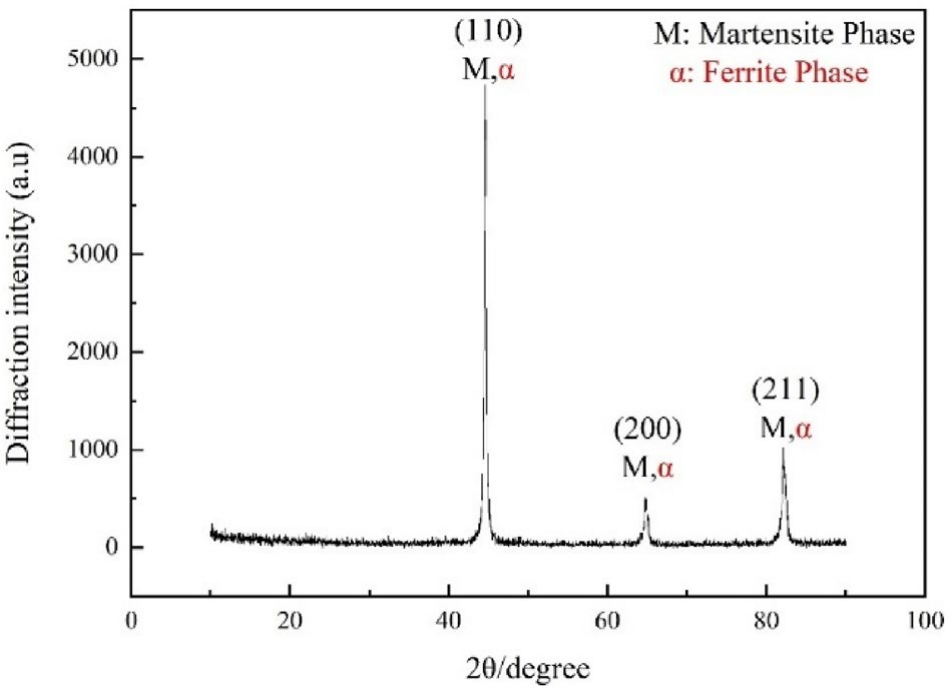
XRD spectra of the treated 35CrMo steel.

As an advanced high-strength steel, the residual stress of constituents affects not only the mechanical properties^44^ but also the dimensional stability^45^ of 35CrMo steel. In this study, the 35CrMo steel is quenched for 40 min at 750 °C and tempered for 120 min at 300 °C to obtain the fine strengthened martensitic phase^46^. The chemical elements of the 35CrMo steel are listed in Table 1. And the microstructure of the treated 35CrMo steel is consisted of the ferrite and martensite^47^, which is confirmed by XRD (CuKα in the D/Max-2550-pc) analysis in Fig. 1.

**Table 1:** Chemical composition of 35CrMo steel (wt%).

Elements	C	Si	Mn	Cr	Mo	Cu	P	S	Ni	Fe
Composition	0.345	0.26	0.55	0.94	0.20	≤ 0.29	≤ 0.035	≤ 0.035	≤ 0.030	Balance

**Figure 2:**
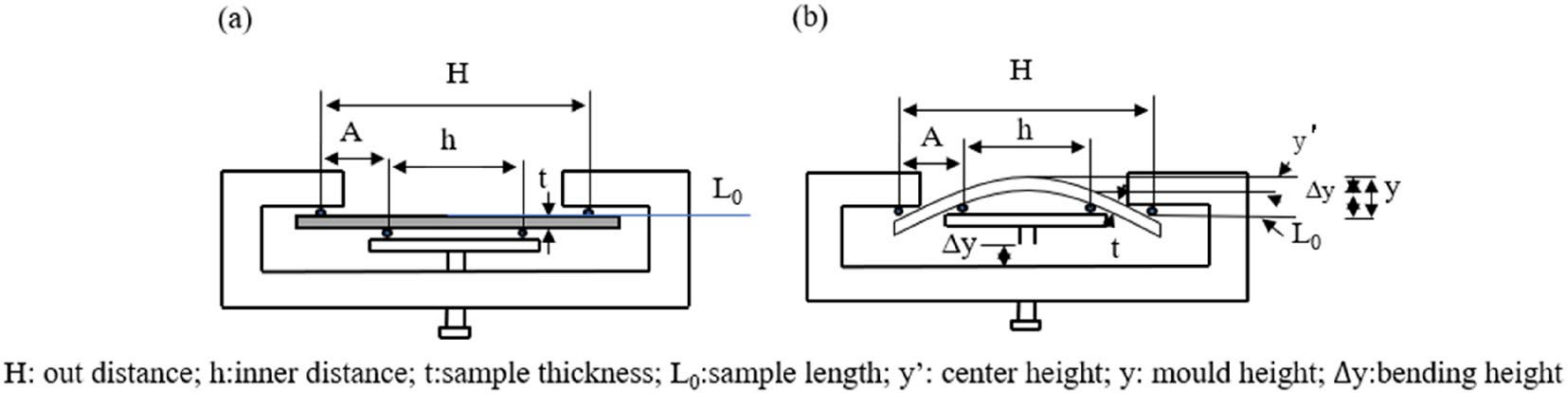
Schematic diagram of four-point bending (**a**) without loading, (**b**) during loading.

And then, the treated 35CrMo steel is cut in the 130 mm × 15 mm × 3 mm sample and the uniaxial stress of the treated 35CrMo steel is carried out by four-point bending^48^ shown in Fig. 2, necessary parameters of.

which are listed in Table 2. As shown in Table 3, macroscopic stresses are applied to the sample during the diffraction strain measurements based on the following, which can refer to the GB/T15970.2–2000.

**Table 2:** Parameters of a four-point bending loading device.

Parameters	H/mm	h/mm	A/mm	E/GPa	t/mm	σ/MPa	∆y/mm
Values	119.43	59.29	31.86	213.00	2.80	Calculated	Measured

**Table 3:** The macroscopic loading by four-point bending for treated 35CrMo steel.

Number	σ/MPa	E/GPa	h/mm	H/mm	A/mm	t/mm	$\Delta y$_1_ /mm
1	174.00	213.00	59.29	119.43	31.86	2.77	0.70
2	199.00	213.00	59.29	119.43	31.86	2.77	0.80
3	223.00	213.00	59.29	119.43	31.86	2.77	0.90
4	273.00	213.00	59.29	119.43	31.86	2.77	1.10
5	323.00	213.00	59.29	119.43	31.86	2.77	1.30

18 $${\sigma }_{\varphi }=\frac{\Delta y}{\frac{\left(3{\mathrm{H}}^{2}-4{\mathrm{A}}^{2}\right)}{12E\mathrm{t}}-\frac{{\mathrm{h}}^{2}}{4E\mathrm{t}}}$$

The diffraction strain measurements of the sample during the four-point bending. In these measurements, the (211) of ferrite is selected as the diffraction crystal plane, and Kα is for the residual-stress tester (x-stress 3000, made in Finland) at 30.0 kV and 6.7 mA for 10 s, following different diffraction angles, 0°, ± 14.5°, ± 20.7°, ± 25.7°, ± 30°.

**Figure 3:**
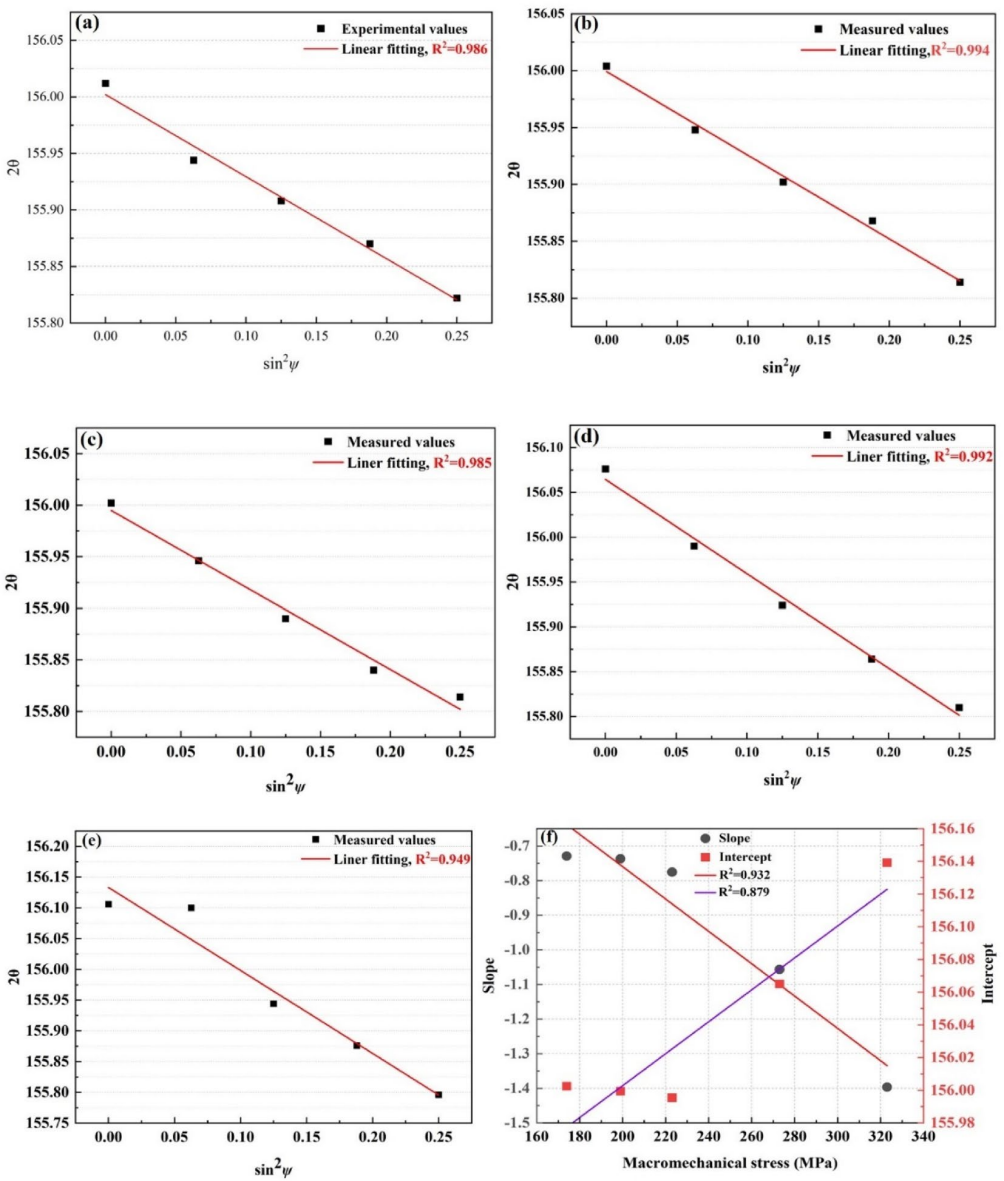
The results of experimental measurements for (211) diffraction plane of ferrite (**a**–**e**) the measurements of diffraction strain at 174 MPa,199 MPa, 223 MPa, 273 MPa, 323 MPa, respectively; (**f**) the diffraction data fitting of (211) diffraction planes of ferrite.

According to the two Tilt method of measurements of residual stress by X-ray diffraction, the diffraction strain ${\varepsilon }_{\varphi \psi }$ at the normal plane is defined as^49^

19 $${\varepsilon }_{\varphi \psi }=\frac{\Delta d}{d}=\frac{1}{2}\mathrm{cot}{\theta }_{0}\cdot \left({2\theta }_{\psi }-{2\theta }_{0}\right)$$

and the relation between the normal stress and determined macroscopic stress is

20 $${2\theta }_{\psi }=-\frac{2\left(1+{\nu }_{hkl}\right)}{{E}_{hkl}}({\sigma }_{\varphi }+{\sigma }_{11}){\mathrm{sin}}^{2}\psi \cdot \mathrm{tan}{\theta }_{0}+\frac{2{\nu }_{hkl}}{{E}_{hkl}}\left({\sigma }_{11}+{\sigma }_{22}+{\sigma }_{\varphi }\right)\mathrm{tan}{\theta }_{0}+2{\theta }_{0}$$

where ${\theta }_{0}$ is the diffraction angle without stress; ${\sigma }_{11}$ and ${\sigma }_{22}$ are normal stress on the sample surface; ${\nu }_{hkl}$ and ${E}_{hkl}$ are the DECs of the constituents; ${\sigma }_{\varphi }$ is the macroscopic stress determined by Eq. (18). The results of the diffraction experiments of ferrite are shown in Fig. 3.

In Figs. 3(a–e), the slope and intercept of the linear fitting of the ${2\theta }_{\psi }\sim {\mathrm{sin}}^{2}\psi$ are $M=-\frac{2\left(1+{\nu }_{hkl}\right)}{{E}_{hkl}}({\sigma }_{\varphi }+{\sigma }_{11})\mathrm{tan}{\theta }_{0}$ and $A=\frac{2{\nu }_{hkl}}{{E}_{hkl}}\left({\sigma }_{11}+{\sigma }_{22}+{\sigma }_{\varphi }\right)\mathrm{tan}{\theta }_{0}+2{\theta }_{0}$, respectively. As a result, the diffraction elastic constants of the (211) diffraction crystal plane are obtained by the following

21 $$ \begin{gathered} \frac{{1 + \nu_{hkl} }}{{E_{hkl} }} = \left( { - \frac{\pi }{{360 \times \tan \theta_{0} }}} \right)\frac{\partial M}{{\partial \sigma_{\varphi } }} \hfill \\ \frac{{\nu_{hkl} }}{{E_{hkl} }} = \left( {\frac{\pi }{{360 \times \tan \theta_{0} }}} \right)\frac{\partial A}{{\partial \sigma_{\varphi } }} \hfill \\ \end{gathered} $$

where $\frac{\partial M}{\partial {\sigma }_{\varphi }}=\hspace{0.17em}-\hspace{0.17em}$0.00467, $\frac{\partial A}{\partial {\sigma }_{\varphi }}=$ 9.76466 × 10^−4^ are the slope of the fitting $M\sim {\sigma }_{\varphi }$ and $A\sim {\sigma }_{\varphi }$ in Fig. 3f, respectively. Thus, the DECs of the (211) diffraction plane of ferrite are *E*_211_ = 250.69 GPa and $\nu$_211_ = 0.261.

The effective elastic constants (EECs) of the treated 35CrMo steel are predicted by this model, the elastic stiffness constants of which are listed in Table 4. Because the carbon atoms are trapped in the octahedral gap between iron atoms^52^, the tetragonal martensite is appeared as supersaturation in the treated 35CrMo steel. The body-centered cubic (bcc) ferrite with three independent elastic stiffness constants is different from the tetragonal martensite with six independent elastic components.

**Table 4:** Elastic stiffness constants of ferrite and martensite.

Constituents	The elastic stiffness constants/GPa						Reference
	${C}_{11}$	${C}_{12}$	${C}_{13}$	${C}_{33}$	${C}_{44}$	${C}_{66}$	
_Ferrite_	243.1	138.1	138.1	243.1	121.9	121.9	Experiment^50^
_Martensite_	235.0	141.0	140.0	267.0	118.0	117.0	Experiment^51^

**Figure 4:**
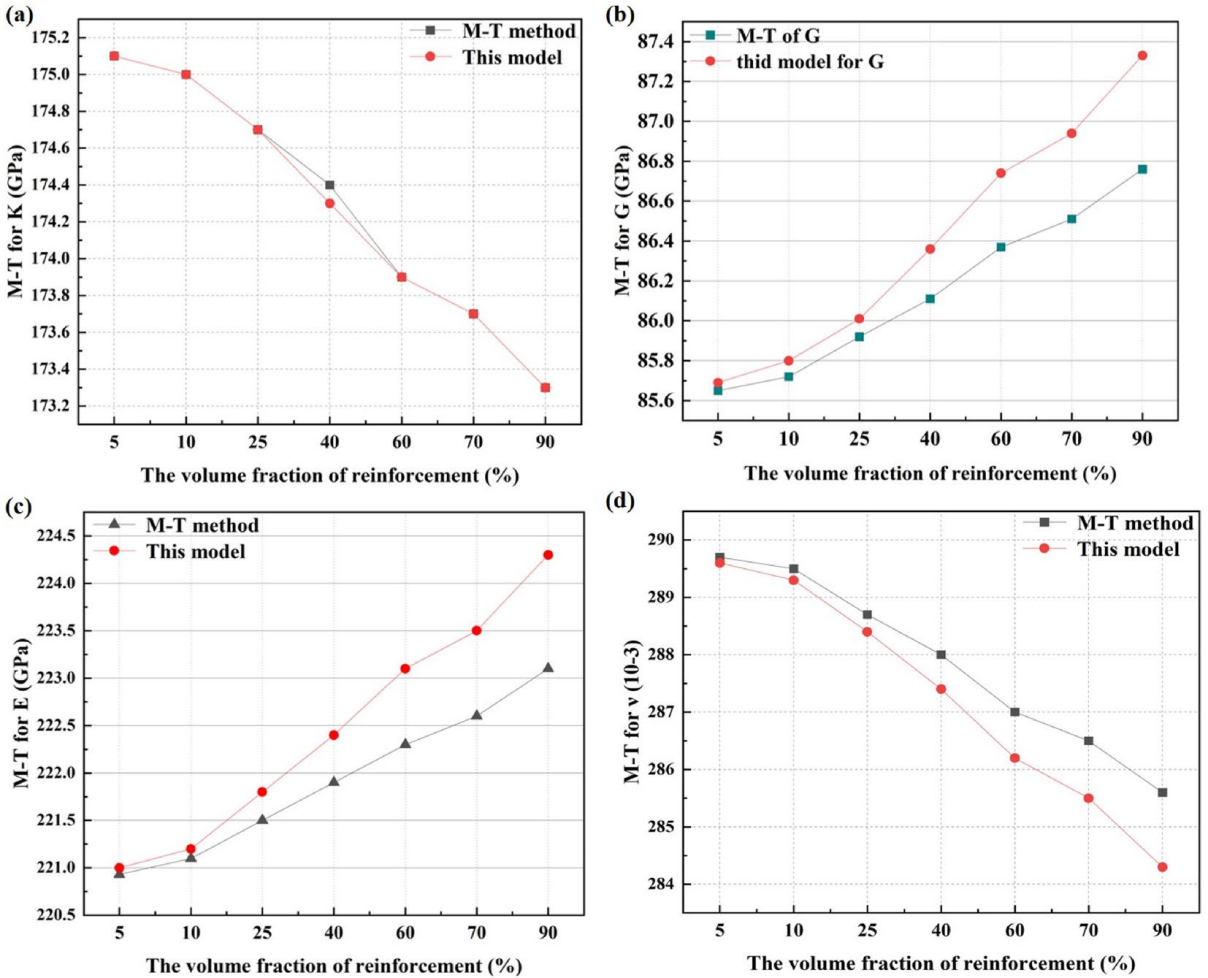
Effect of reinforcement on EECs (**a**) $\overline{K }$, $\overline{G }$ and $\overline{E }$; (**b**)$\overline{\nu }$.

In Table 5, the predicted EECs of both the treated 35CrMo steel and its constituents by this model agree better with the experimental values than the other micromechanical models, including the effective elastic modulus $\overline{E }$, effective elastic bulk modulus $\overline{K }$, effective shear modulus $\overline{G }$ and effective Poisson's ratio $\overline{\nu }$. In this model, the effective bulk modulus $\overline{K }$ and shear modulus $\overline{G }$ are predicted by the Eq. (9), and the effective bulk modulus $\overline{E }$ and $\overline{\nu }$ are predicted by the Eq. (3). The other analytical details of micromechanical models can refer to corresponding literature. It is noted that the predicted the effective elastic constants (EECs) by this micromechanical model and Kröner model^41^ are within in the Reuss-Voight limit, which indicates that the interaction between crystals cannot be ignored. However, the accuracy of this model is better than the Kröner model^41^ for that the interaction between isotropic material is not enough to reveal the influence between anisotropic constituents on the composite deformation behavior. As for composites, the prediction of the EECs by the micromechanical model is almost identical to the Mori–Tanaka (M-T) method^35^. For example, the predicted EECs of the treated 35CrMo steel are slightly higher than theexperiments except the $\overline{G }$ with the maximum 4.64% bias. Considering objective differences between the model assumption and real situation, it is credible to predict the effective elastic response of the treated 35CrMo steel by this micromechanical model. Furthermore, the effect of ferrite on the EECs of the treated 35CrMo steel is also evaluated in Fig. 4.

**Table 5:** Calculated and experimental values of effective elastic constants.

Materials	Effective elastic constants				Reference
	$\overline{E }$/GPa	$\overline{K }$/GPa	$\overline{G }$/GPa	$\overline{\nu }$	
Ferrite	224.70	173.10	87.50	0.284	Experiment^50^
	223.40	173.10	86.90	0.285	This study
	207.40	173.10	79.74	0.300	Reuss model^39^
	239.10	173.10	94.14	0.270	Voight model^40^
	236.2	173.10	92.8	0.273	Kröner model^41^
Martensite	220.83	175.21	85.59	0.290	this study
	204.06	174.83	78.16	0.290	Reuss model^39^
	234.21	175.44	89.70	0.278	Voight model ^40^
	213.75	175.44	82.40	0.297	Kröner model ^41^
35CrMo steel	213.00	169.06	82.11	0.291	experiment ^53^
	221.78	174.67	86.07	0.288	this study
	221.45	174.68	85.92	0.289	M-T method^35^

**Figure 5:**
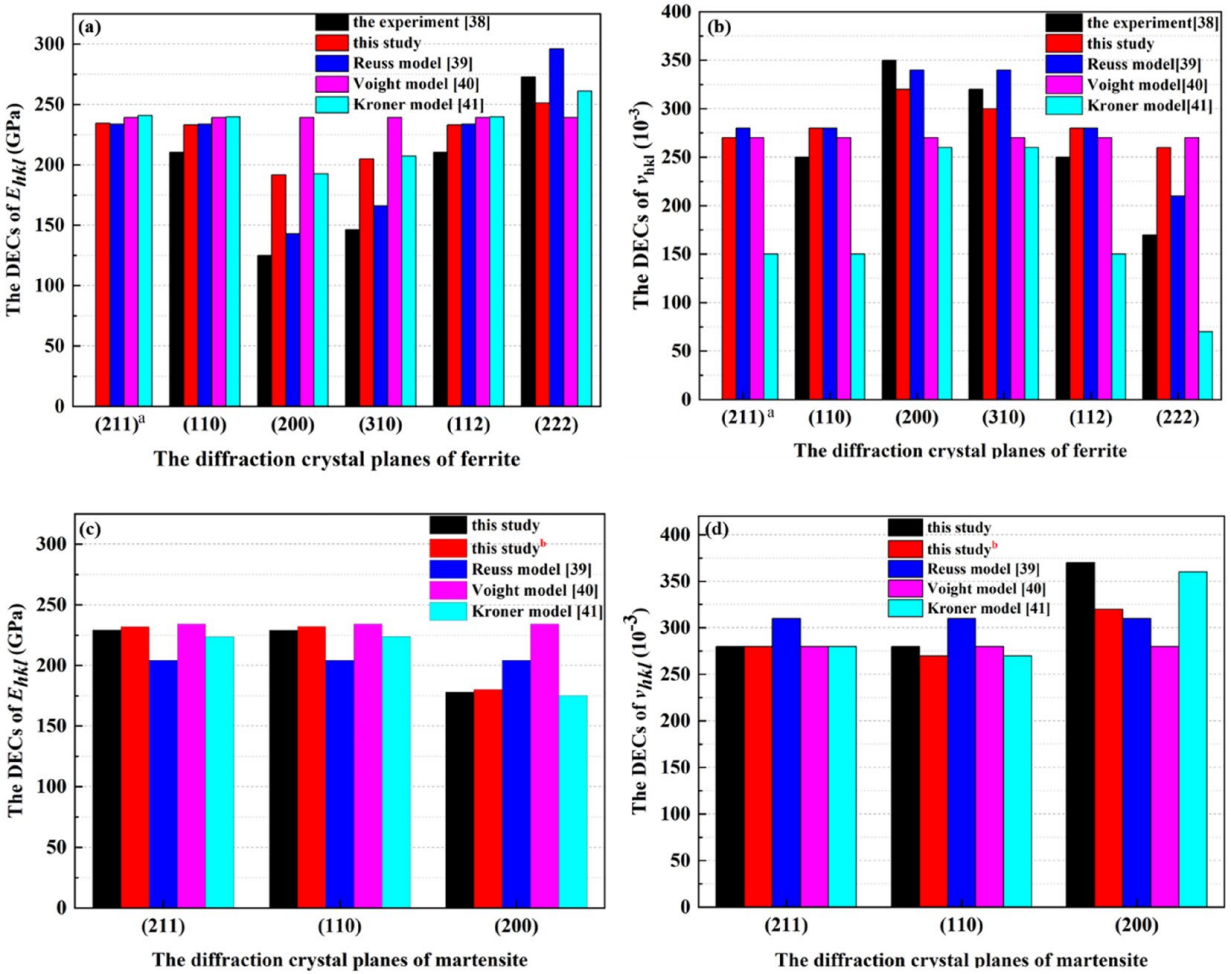
The DECs of constituents (**a**) *E*_*hkl*_ of the ferrite; (**b**) *v*_*hkl*_ of the ferrite; (**c**) *E*_*hkl*_ of the martensite; (**d**) *v*_*hkl*_ of the martensite.

As Fig. 4 shows, the effective elastic constants (EECs) of the treated 35CrMo steel predicted by the micromechanical model are almost identical to that of the M-T method^35^. The EECs of the treated 35CrMo steel hardly change with the ferrite, including the effective elastic bulk modulus $\overline{K }$ (from 175.1 to 173.3 GPa by M-T method and this model), the effective elastic bulk modulus $\overline{G }$ (from 85.65 to 86.76 GPa by M-T method, from 85.69 to 87.33 GPa for this model), the effective elastic bulk modulus $\overline{E }$ (from 220.93 to 223.1 GPa by M-T method, from 221.0 to 224.3 GPa for this model), the effective Poisson's ratio $\overline{\nu }$ (from 0.290 to 0.286 by M-T method, from 0.290 to 0.284 for this model), which means that relatively small elastic difference shown in Table 5 between the ferrite and martensite will not significantly affect the elastic properties of the treated 35CrMo steel. In other words, the reinforcement effect on the material depends on the elastic properties of the constituents and the elastic anisotropy of materials is not obvious resulted by relatively small elastic difference of constituents. Although both the M-T method and this model are equally applicable in simulating the effective elastic response of the materials defined by the Eq. (2), the interaction between the constituents is not considered by the M-T method shown as Eq. (13). As mention above, purpose of this improved multiscale constitutive model is the correlation mechanism between the macroscopical elastic deformation of the whole material and the internal mechanic of the constituents. And then, the comparison between the diffraction elastic constants of the constituents of the treated 35CrMo steel predicted by different model is shown in Fig. 5.

In Figs. 5a, b, the diffraction crystal plane of ferrite of the constituents of the treated 35CrMo steel are respectively denoted by the superscripts “a” and “b”, in order to quantitatively analyze the difference of diffraction elastic constants predicted by Reuss^39^, Voight^40^, and Kröner^41^ models. Compared with the experimental measurements E_211_ = 250.69GPa and $\nu$_211_ = 0.261 in this study, the accuracy of Reuss^39^, Voight^40^, and Kröner^41^ model is not acceptable, especially the maximum numerical error of 42.3% of $\nu$_211_ from Kröner model^41^, which demonstrates the significance of the correlation mechanism between the macroscopical elastic deformation of the whole material and the internal mechanic of the constituents. It is more feasible of the micromechanical model to predict the diffraction elastic constants (DECs) of ferrite, except for the (200) and (310) diffraction crystal planes, which confirms the generalization ability of this improved model. In addition to ferrite, the DECs of martensite predicted by different models are shown in Figs. 5c, d. As observed, the elastic anisotropy *E*_*hkl*_ of martensite is not obvious as same as that of ferrite, no matter for the constituents of treated 35CrMo steel and single-phase martensite, which may illustrate the reason that the Reuss^39^, Voight^40^, and Kröner^41^ model are generally used for the quantitative analysis of residual stress in practice.

In essence, the Voight model is limited in inhomogeneous strain so that both the *E*_*hkl*_ and *ν*_*hkl*_ of constituents are isotropic^54^. Based on the uniform stress distribution in the polycrystalline, analytical prediction of the diffraction elastic constants (DECs) of Reuss model merely provides the boundary of elastic properties, which is resulted by the inherent difference of average strain in parallel and normal to the uniaxial stress^54^. Although the crystal interaction is significant for predicting DECs the constituents, the reliability of the Kr $\ddot{\mathrm{o}}$ ner model in simulating the micromechanical elastic response of the complex composite is unsatisfactory shown in Fig. 5. As described the Eq. (10) and (17), the micromechanical elastic response of the constituents to the macroscopic stress is reflected by the DECs, which means multiscale correlation of elastic response should be considered in predicting the elastic properties of materials. Consequently, the multiscale correlation between the effective elastic response of material and micromechanical elastic response of constituents made this developed model better generalization ability.

**Figure 6:**
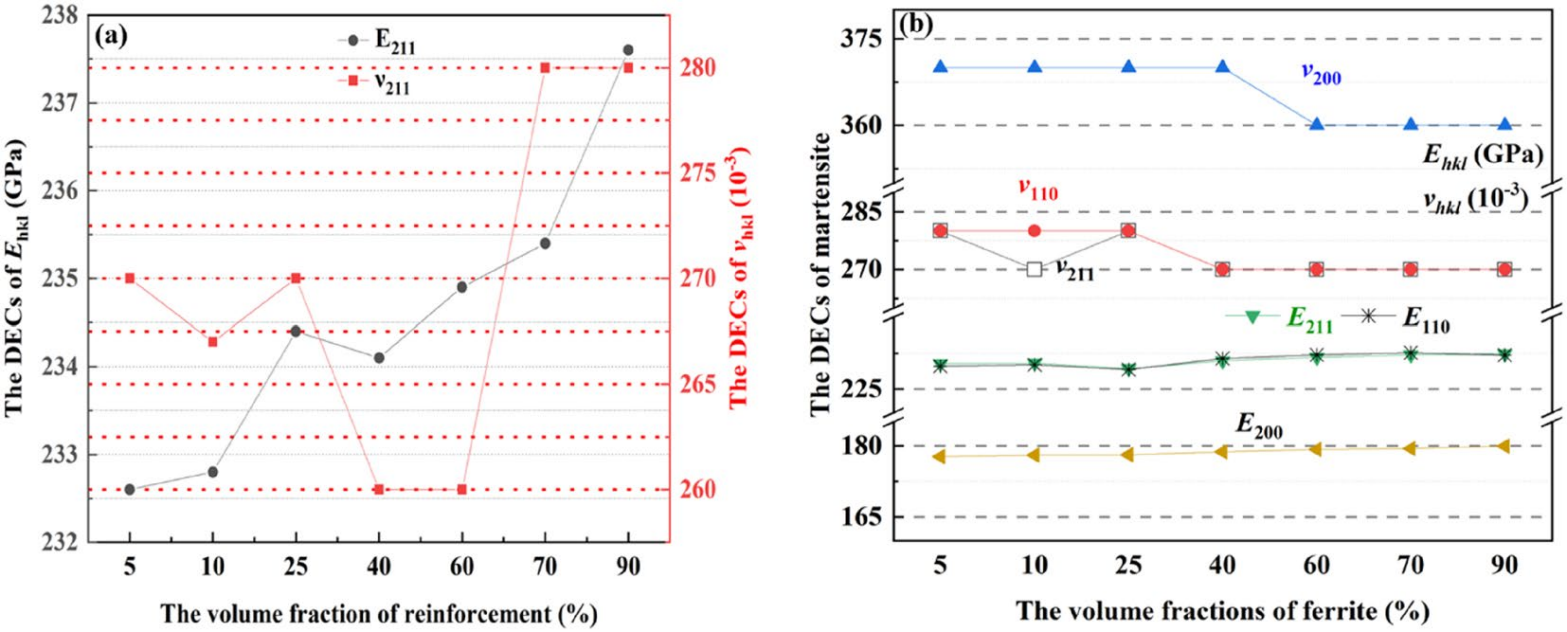
DECs of constituents as a function of reinforcements (**a**) DECs of the ferrite; (**b**) DECs of the martensite.

Furthermore, the influence of ferrite on the diffraction elastic constants (DECs) predicted by this micromechanical model is shown in Fig. 6. As Figs. 6a, b show, the effective elastic modulus of ferrite and martensite in Table 5 are respectively 224.7GPa and 220.83GPa, slight difference of which made DECs indiscernible changes as ferrite. Considering the multiscale correlation mechanism between effective elastic response of the treated 35CrMo steel and micromechanical elastic response of the constituents, the effective elastic constants of the treated 35CrMo steel do not vary significantly with ferrite as shown in Fig. 4, so do the DECs of constituents.

### Conclusion

The multiscale correlation constitutive models of the elastic response to the macroscopic stress are established in this study. Based on the above results, the following conclusions can be drawn.

1. Following the contents of this micromechanical model, both the predicted effective elastic constants (EECs) of whole material, including the effective elastic modulus $\overline{E }$, effective Poisson's ratio $\overline{\nu }$, effective bulk modulus $\overline{K }$, effective shear modulus $\overline{G }$, and diffraction elastic constants of the constituents (DECs), ${\nu }_{hkl}$ and ${E}_{hkl}$, agree well with the experiments.
2. Simple in-situ X-ray diffraction consisted of the four-point bending is successfully performed for the measurements of ${E}_{211}$ and ${\nu }_{211}$ of the ferrite, which further indicates the accuracy and reliability of the nondestructive measurement of residual stress.
3. The multiscale simulation for elastic response reveals the significance of the association mechanism between the whole material and the constituents.

## Data Availability

The datasets generated during the current study are available from the corresponding author on reasonable request.
